# Exploring a Tomato Landraces Collection for Fruit-Related Traits by the Aid of a High-Throughput Genomic Platform

**DOI:** 10.1371/journal.pone.0137139

**Published:** 2015-09-22

**Authors:** Adriana Sacco, Valentino Ruggieri, Mario Parisi, Giovanna Festa, Maria Manuela Rigano, Maurizio Enea Picarella, Andrea Mazzucato, Amalia Barone

**Affiliations:** 1 Department of Agricultural Sciences, University of Naples Federico II, Portici, Italy; 2 Consiglio per la ricerca in agricoltura e l’analisi dell’economia agraria, Centro di ricerca per l’Orticoltura, Pontecagnano, Italy; 3 Department of Science and Technologies for Agriculture, Forestry, Nature and Energy (DAFNE University of Tuscia, Viterbo, Italy); University of Tsukuba, JAPAN

## Abstract

During its evolution and domestication *Solanum lycopersicum* has undergone various genetic ‘bottlenecks’ and extreme inbreeding of limited genotypes. In Europe the tomato found a secondary centre for diversification, which resulted in a wide array of fruit shape variation given rise to a range of landraces that have been cultivated for centuries. Landraces represent a reservoir of genetic diversity especially for traits such as abiotic stress resistance and high fruit quality. Information about the variation present among tomato landrace populations is still limited. A collection of 123 genotypes from different geographical areas was established with the aim of capturing a wide diversity. Eighteen morphological traits were evaluated, mainly related to the fruit. About 45% of morphological variation was attributed to fruit shape, as estimated by the principal component analysis, and the dendrogram of relatedness divided the population in subgroups mainly on the basis of fruit weight and locule number. Genotyping was carried out using the tomato array platform SolCAP able to interrogate 7,720 SNPs. In the whole collection 87.1% markers were polymorphic but they decreased to 44–54% when considering groups of genotypes with different origin. The neighbour-joining tree analysis clustered the 123 genotypes into two main branches. The STRUCTURE analysis with K = 3 also divided the population on the basis of fruit size. A genomic-wide association strategy revealed 36 novel markers associated to the variation of 15 traits. The markers were mapped on the tomato chromosomes together with 98 candidate genes for the traits analyzed. Six regions were evidenced in which candidate genes co-localized with 19 associated SNPs. In addition, 17 associated SNPs were localized in genomic regions lacking candidate genes. The identification of these markers demonstrated that novel variability was captured in our germoplasm collection. They might also provide a viable indirect selection tool in future practical breeding programs.

## Introduction

The cultivated tomato (*Solanum lycopersicum* L.) is a major vegetable crop grown worldwide, from the tropics to within a few degrees of the Arctic Circle [1] with a worldwide production of about 164 million tonnes in the 2013 [2]. Despite its economic importance, some essential aspects of relationships among species related to the cultivated tomato, cultivars and landraces establishing its wide array have yet to be clarified.

A commonly accepted hypothesis for the domestication of cultivated tomato is that the *S*. *lycopersicum* subsp. *cerasiforme* (Dunal) spread as a weed from the Andean region to Mexico, where it was domesticated [3]. The domesticated tomato was taken to Europe in the sixteenth century [4] and it was then disseminated to many areas of the world, where selection for fruit shape and size played a key-role in the morphological diversification of this species [5]. During its evolution and domestication *S*. *lycopersicum* has undergone various genetic ‘bottlenecks’ imposed by self-pollination, founder effects, artificial and natural selection, in addition to extreme inbreeding of limited genotypes, particularly in Europe and North America [6]. In Europe, the tomato has been most successful in the Mediterranean countries, particularly in Italy and Spain [7]. In these countries, *S*. *lycopers*i*cum* found a secondary centre for diversification [8], which resulted in a wide array of fruit shape variations including round, obovoid, long, heart, blocky and even bell pepper-shaped fruits. This variation has given rise to a range of landraces that have been cultivated for centuries and many of these are still commonly found at local markets [7]. Characterized by a good stress tolerance and local adaptability despite their lack of pathogen resistance genes, landraces still represent a reservoir of genetic diversity especially for traits of interest such as abiotic stress resistance and high fruit quality [9]. For these reasons heterogeneous landrace populations are very important genetic resources and have been, and will continue to be, used in plant breeding schemes. Genetic profiles of tomato landraces are clearly different from those of modern tomato varieties [7,10–11]. Using morphological/agronomical traits, biochemical characteristics and molecular markers, significant levels of phenotypic and genetic diversity have been observed [11–17]. However, information about the variation present among tomato landrace populations is still limited. Understanding genetic diversity in traditional tomato accessions is therefore important not only for germplasm management and valorisation but also for crop breeding. Unravelling genetic variability in cultivated landraces will shed additional light on the developmental regulation of fruit shape and size and will also help to identify novel alleles and/or haplotypes to improve productivity, adaptation, quality and nutritional value [18]. In addition, exploiting broad genetic variability in association mapping studies offers the opportunity for searching genotype-phenotype correlations among unrelated individuals, and for identifying superior alleles. The genome wide association strategy (GWAS) often requires a large number of markers for genotyping the germplasm collection under study. Currently, the availability of cost-effective and fast genotyping assays has made single nucleotide polymorphisms (SNPs) the markers of choice for genome-wide genetic analyses encouraging the study of large germplasm collections. As for tomato, in addition to other high-throughput platforms used to explore polymorphisms at genome-wide level [19–21], a SolCAP chip based on ILLUMINA Infinium Technology has been developed [22,23] to interrogate about 8,000 SNPs in different germplasm collections.

In the present study, in order to investigate genetic variation in the tomato genome to find new favourable alleles for tomato breeding, a tomato collection of 123 genotypes was analyzed using the SolCAP SNP array. The main goal of our work was to characterise at morphological and molecular level the selected tomato collection with special emphasis on fruit morphology. Specifically we wanted to 1) explore the genetic diversity available in our wide germplasm collection, 2) test the established collection for association mapping analysis.The study evidenceda wide genetic variability in our tomato collection,whichturned out to be suitable forsuccessfully GWAS approaches. We were able to identify 36 novel markers to target the phenotypic variability of 15 traits, mostly relating to fruit morphology.

## Materials and Methods

### Plant materials

A germplasm collection of 123 genotypes was established by selecting tomato accessions worldwide with the aim of capturing a wide diversity in our panel. This collection enhances the variability of one population made of 90 genotypesand previouslyused in our laboratory for GWAS for fruit quality related traits [24].The germplasm evaluated here consisted of 61 Italian landraces (IL), 26 American landraces (AL), 15 landraces coming from geographical areas different from Italy and South/Central America (OL), 19 cultivars (CV), and two wild species (WS). The latter were *Solanum pimpinellifolium* accession LA1579 and *Solanum*. *lycopersicum* var. *cerasiforme* accession LA1310. The material is listed in S1 Table. Accessions were obtained from the Tomato Genetic Resource Center (TGRC, Davis, USA), the Centre for Genetic Resources (CGR, Wageningen, The Netherlands), the USDA, the Campania Region Agricultural Unit, and from tomato germplasm collections held at the CRA-Vegetable Crop Research Centre, at the University of Tuscia and at the University of Naples by the authors. Plants were grown according to a completely randomized block design with three replicates (10 plants/replicate), in an experimental field located in Southern Italy (Pontecagnano, Campania Region) with standard agronomic practices. No specific permissions were required for field activities because our experimental field was included in the facilities of the Council for Agricultural Research and Economics CRA-ORT Centro di Ricerca per l'Orticoltura/Vegetable Crop Research Centre who was partner in the project. We also confirm that the field studies did not involve endangered or protected species. Morphological data were recorded at different developmental stages and young leaves were collected from one representative plant *per* each accession, then stored at -80°C for DNA extraction.

### Morphological characterization

For each accession, 18 morphological traits were evaluated, mainly related to the fruit (S1 Table). At 80 d from sowing, growth habit (GH), plant height (PH), inflorescence type (IT) and green shoulder (GS) were measured or scored. Ten ripe fruits *per* replica *per* genotype were evaluated for fruit colour (FC), fruit flesh colour (FLC), polar diameter (PD), equatorial diameter (ED), stem-end shape (SES), blossom-end shape (BES), number of fruit locules (LN), pericarp thickness (PT), puffiness (PUF), fruit-shape index (FS = PD/ED), fruit weight (FW), fruit-shape longitudinal section (FSL), and fruit-shape cross section (FSC). In addition, the fruit-shape (FS = PD/ED) and pericarp-thickness index (PI = PT/((PD+ED)/2) were calculated from raw data.

The morphological evaluations were carried out following the descriptors indicated by [13] for 12 traits (GH, PH, IT, GS, FC, PD, ED, PT, PUF, FS, FW and PI), while for five traits (SES, BES, LN, FSL and FLC) the instructions of DUS Test were adopted (CPVO-TP/044/4 Final). To better evaluate the phenotypic variability of the collection, for FSL, BES and SES, the scales were further modified, as indicated in S1 Table. Finally, FSC trait was recorded following the IPGRI/Biodiversity protocol (Descriptors for Tomato, 1996).As for PH, quantitative data were split into three classes with class size corresponding to (max value—min value)/3. A Spearman’s rank correlation coefficient was calculated among all the variables. Factor analysis including Kaiser-Meyer-Olkin (KMO) and Bartlett's tests was performed using SPSS software 21.0. The tomato collection was clustered into a dendrogram of relatedness using the ggplot2 package in R version 2.15.0 software [25].

### Genotypic characterization

Genomic DNA was extracted from100 mg of frozen leaves following a modified protocol of the cetyltriethylammoniumbromide (CTAB) extraction method described by [26]. DNA quantity and quality were evaluated by a Nano Photometer^TM^(Implen) at 260/280 and 260/230 OD ratios. Genotyping was carried out using the tomato array platform SolCAP developed in the framework of the Solanaceae Coordinated Agricultural Project from NIFA/USDA and based on the ILLUMINA Infinium Technology [23]. The Illumina assay and subsequent SNP calling were performed as previously described in [24]. Data were analyzed and markers with more than 10% missing genotypes were removed. A neighbour-joining tree was generated using the TASSEL software [27]. The genotypic data were subjected to different within and among groups genetic diversity measures, such as Major Allele Frequency (MAF), levels of observed (H_o_) and expected (H_e_) heterozygosity (Gene diversity) and Polymorphism Information Content (PIC). All these calculations were performed using PowerMarker software 3.25 [28] on six different datasets: five included SNPs revealed on groups AL, CV, IL, OL, WS, and one included SNPs revealed on the complete set of genotypes. Also, linkage disequilibrium (LD) decay of the entire tomato collection was calculated for each chromosome as reported by [24].

To assess the genetic relationships of the investigated genotypes the population structure was determined by using STRUCTURE 2.3.3 software [29], with no *a priori* information regarding population origin. The degree of admixture was estimated by setting for both burn-in period and Markov Chain Monte Carlo iterations a value of 100.000 for each run. Ten independent runs across a range of K values (K = 1–12) were made. The best number of clusters (K) was obtained using STRUCTURE HARVESTER program [30] based on the method of Evanno [31].

### Genome-wide association analysis

Associations between genotypes and phenotypes were calculated for all 18 morphological traits, excluding the two wild accessions given their high phenotypic differences compared to the whole collection. Associations were detected using the mixed linear model (MLM) implemented in TASSEL program, which accounts for kinship (K matrix) and population structure (Q matrix) matrices. Significant levels of association (p = 0.05) were estimated considering an adjusted *P* value of 4.1x10^-4^ after the Bonferroni correction. To ascertain the effectiveness of our association analysis, genes controlling fruit morphology traits and plant architecture in tomato were selected as candidate genes (CGs). CGs were identified both from the SOL genomics network [32] and from literature. To this end we focused the research on key-words regarding fruit shape, size,colour, plant habit and also floral meristem and ovary size due to the impact of this last traits on the fruit size and morphology. Finally, a physical map of the tomato genome showing the position of the candidate genes and the SNP markers significantly associated with the traits was constructed using the Map Chart software 2.2 [33].

## Results

### Morphological analysis

**Fig 1:**
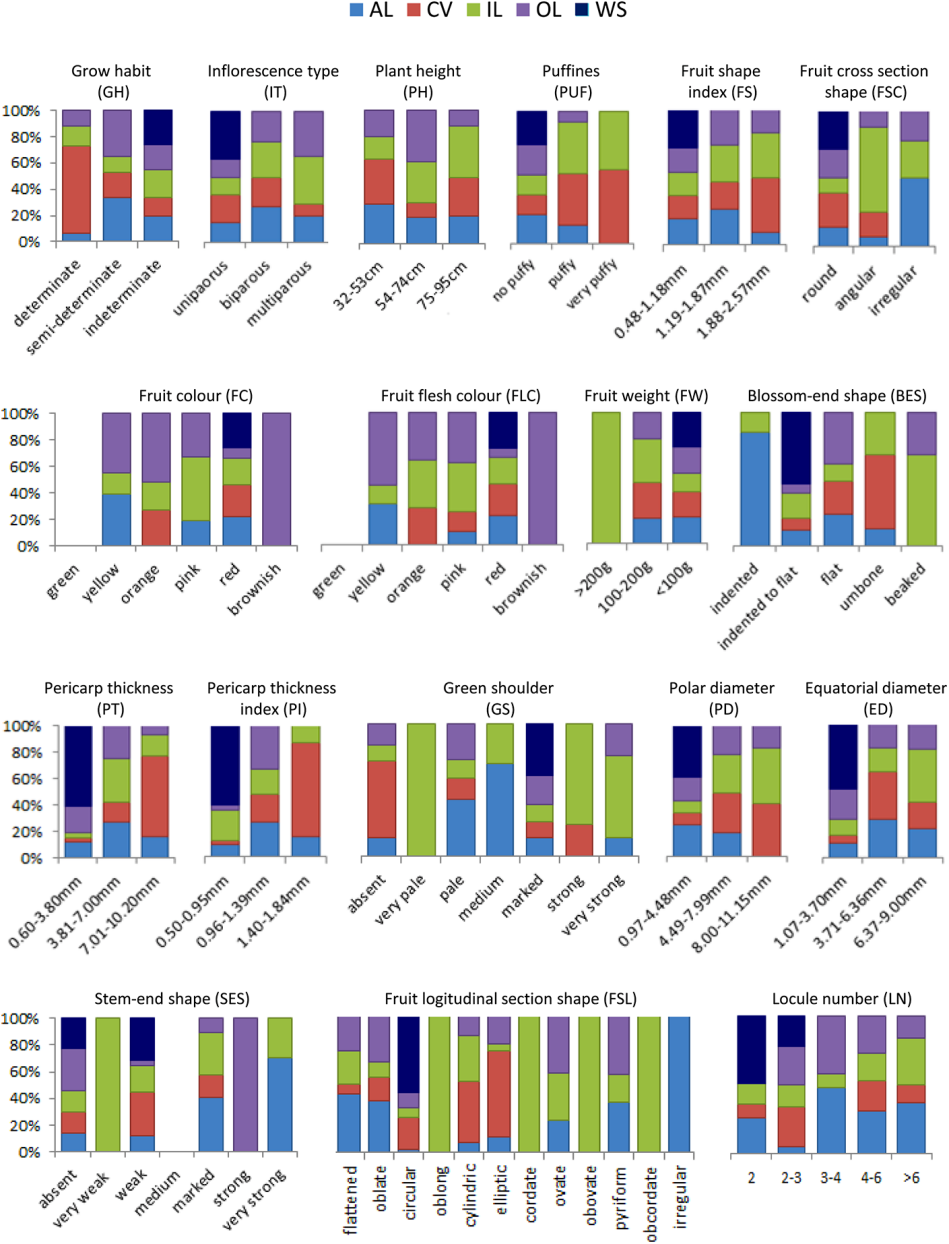
Variation of morphological traits. Distribution of the 18 morphological traits throughout the groups in which the different genotypes have been divided based on their origin (AL = American Landraces; CV = Cultivars; IL = Italian Landraces; OL = Other Landraces; WS = Wilde Species). Each chart represents a different trait.

Various traits were evaluated to phenotype our collection, detailed data are reported in the S1 Table. The variation of the morphological data through the groups in which the tomato collection has been divided is shown in Fig 1. Among the 18 traits measured, two were related to plant growth and one to reproductive structures. In particular, as for growth habit (GH) our collection mainly consisted of indeterminate genotypes (95 out of 123, 77.2%) whereas the others were split between determinate and semi-determinate habit (14 genotypes, each). Plant height (PH) was measured and genotypes were consequently classified in three groups: the majority (57, 46.3%) belongs to group 1 (32–53 cm) or group 2 (54–74 cm) with 43 genotypes(34.9%). The inflorescence type (IT) was uniparous in 51 (41.4%) genotypes, biparous in 52 (42.3%) and multiparous in 20 (16.3%).

**Fig 2:**
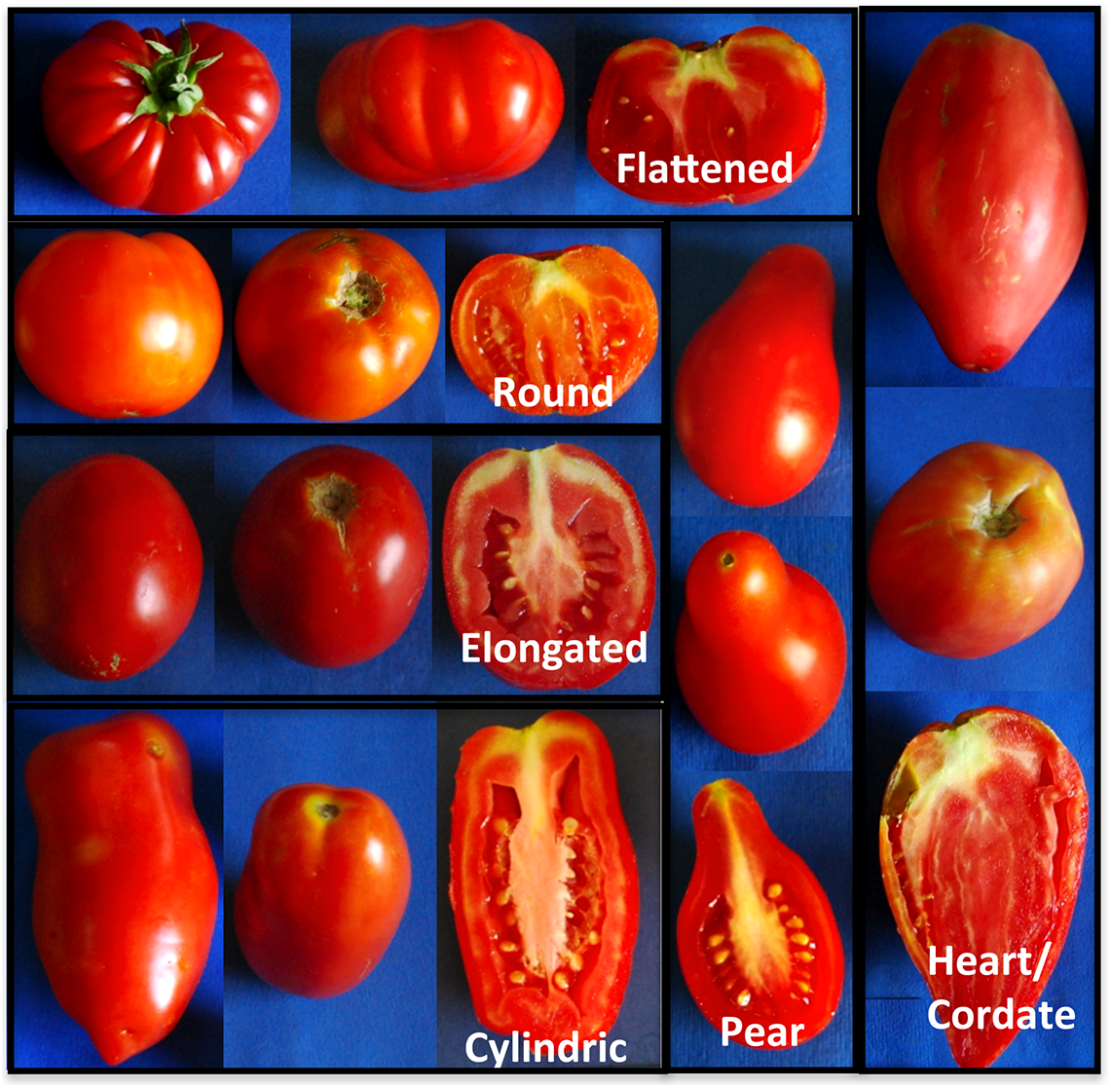
Diversity in tomato fruit shapes. Tomato fruit shape categories adapted from the IPGRI/Biodiversity protocol (1996).

**Fig 3:**
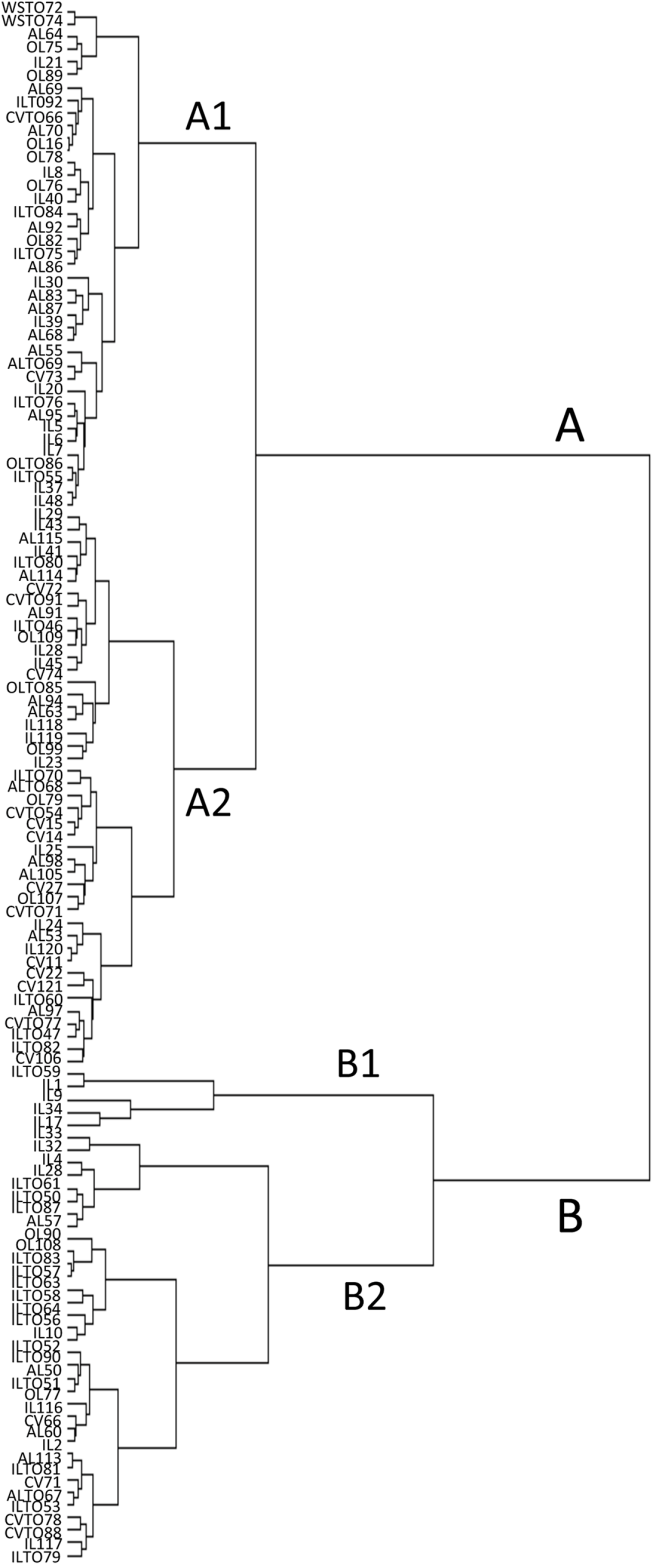
Hierarchical clustering analysis. Dendrogram of relatedness of the tomato genotypes based on morphological traits.

As for traits related to fruit colour and fruit flesh colour, most genotypes (96) had red fruit, 26 genotypes had fruit that varied from yellow, to orange or pink, and five had brownish fruit. Some genotypes exhibited an absent (20) or very week (31) green shoulder, whereas a medium intensity or a strong/very strong intensity was recorded for 46 and 26 genotypes, respectively. Several traits were measured to determine the fruit shape, such as polar (PD) and equatorial (ED) diameter, stem- and blossom-end shape (SES, BES), longitudinal and cross section (FSL, FSC), puffiness (PUF). Altogether, including the number of fruit locules (LN), these traits contributed to determine six different fruit-shapes (Fig 2). Many genotypes (33%) had a flattened shape with fruit of small (<100gr) or medium (100–200gr) size. Also, the elongated shape was represented in about 20% of genotypes, all exhibiting a small sized fruit.The Spearman’s rank correlation coefficients calculated between pairs of variables (S2 Table) evidenced significant values (p = 0.05) higher than 0.6 for LN *vs*. ED (0.795), SES *vs*. ED (0.719), LN *vs*. SES (0.749), FW *vs*. LN (0.678), and FS *vs*. BES (0.675). Extremely high correlation values were observed for FC and FLC (0.98), as expected, and between FW and ED (0.913). Significant negative correlations were observed for FS *vs*. LN (-0.714), and FS *vs*. SES (-0.666). The KMO (>0.7) and Bartlett’s tests (p = 0.000) indicated the suitability of our data for structure detection. Principal component analysis (PCA) reduced the 18 morphological characters to two principal components, which accounted for 46% of the total variation. The first component (PC1) explained 29.95% of the variation and was mainly associated to LN, SES, ED, FSC, FW and IT. The second component (PC2) explained 16.29% of the total variation and was basically defined by PD and PT (S1 Fig). The majority of OLs and CVs resided in the lower quadrants while most ALs in the left quadrants of the chart, on the contrary ILs were spread along the whole chart, and WSs were clearly separated. In any case, no specific group based on accession origin clustered in any quadrant and the collection was evenly distributed among them.Based on morphological characterization, the whole collection was clustered into a dendrogram of relatedness (Fig 3), which identified two main groups. The first group (A) included 84 genotypes with 34 ILs, 21 ALs, 15 CVs, 12 OLs, and the two wild species. All genotypes in this group exhibited a fruit weight ≤100gr. In the sub-group A1 most genotypes except two (AL68 and AL83) had fruit with low LN (one to three locules), whereas in the sub-group A2 a higher number of genotypes (14 out of 44) showed fruit with high LN. Cluster B (39 genotypes as a whole) principally included genotypes with fruit weight >100 gr and mainly consisted of ILs (27 out of 39, 69%). In this group all genotypes exceptfour (CVTO78, CVTO88, IL117, ILTO79) exhibited fruit with high LN.

### Molecular analysis

#### Genetic diversity and population structure

The 123 genotypes were screened by 7,720 SNPs, among which 7,672 were successfully scored. As a whole, 4,763 SNPs exhibited minor allele frequency lower than 0.05. Table 1 reports the descriptive statistics of all SNPs analyzed. This evidenced that high values of the major allele frequency were observed in most cases (it was always higher than 0.8 except than in the WS group). Moreover, in all groups the average of the observed heterozygosity (ranging from 0.01 in the CV group to 0.06 in the WS group) was lower than the expected heterozygosity, as estimated by the gene diversity index, which ranged from a minimum of 0.095 (ILs) to a maximum of 0.266 (WSs). Finally, the PIC index varied from 0.079 for ILs to 0.205 for WSs. As a whole, among the 7,672 SNPs analyzed, the polymorphic SNPs in the collection were 87.1%, but they decreased to 49.0%, 53.7%, 53.0%, 44.1% and 54.9% in the AL, CV, IL, OL and WS groups, respectively, thus evidencing that in each group a number of SNPs did not segregate with respect to other groups.

**Table 1:** Descriptive statistics for the genetic diversity within groups.

Group	Sample Size	MAF^1^	(H_e_)^2^	(H_o_)^3^	PIC^4^
AL	26	0.918	0.1146	0.01486	0.0966
CV	19	0.8847	0.1595	0.01274	0.1315
IL	61	0.9305	0.0951	0.01714	0.0798
OL	15	0.9037	0.1359	0.01763	0.1133
WS	2	0.7475	0.2665	0.06381	0.2049
Total	123	0.9155	0.1249	0.01678	0.1061

^1^Major Allele Frequency.

^2^Expected heterozygosity.

^3^Observed heterozygosity.

^4^Polymorphism Information Content.

**Fig 4:**
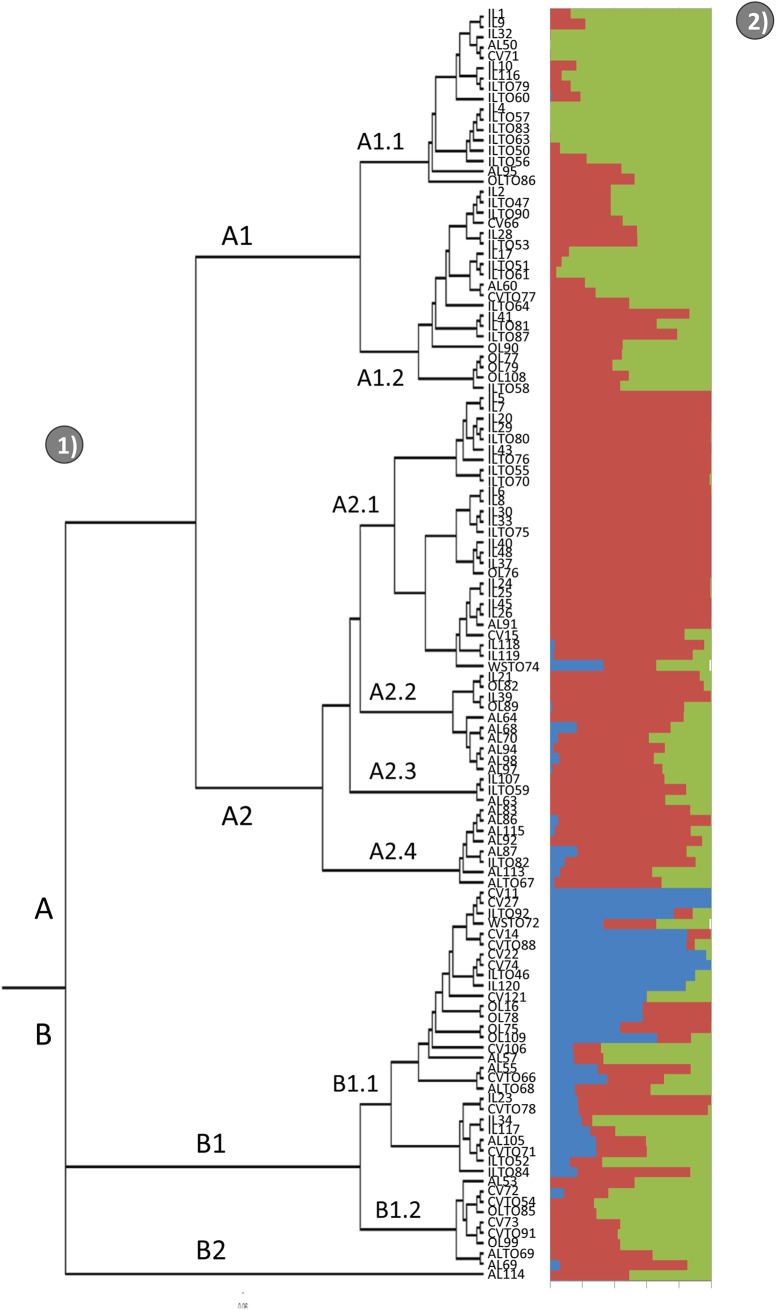
Genetic structure of the tomato collection. 1) Neighbor-joining tree analysis generated using TASSEL software; A (A1-A2.4) and B (B1-B2) stands for branch or cluster. 2) Population stratification inferred for K = 3. Each bar stands for a genotype, which is partitioned into color segments that represent the estimated membership fraction in the K cluster.

The population stratification of our tomato collection was investigated without introducing any *a priori* classification. The neighbour-joining tree analysis clustered the 123 genotypes into two main branches (A and B) (Fig 4). Branch A comprises 85 genotypes, branch B consists of 38 genotypes. Branch B immediately differentiates into sub-groups B1 and B2, the latter only including genotype AL114, a landrace from Chile. In particular, most of the ILs (49 out of 63) clustered in the upper part of the tree (branch A1.1, A1.2, A2.1, with 13, 13 and 23 ILs, respectively); ALs mainly clustered in the middle (14 out of 26 in branches A2.2, A2.3, A2.4) and CVs at the bottom (14 out of 19 in branch B1.1); finally the OLs spread more or less uniformly among the different branches. Alongside the tree analysis, a model-based clustering method implemented in STRUCTURE was performed. The STRUCTURE analysis resulted in a prediction for K of either 3 or 11. When K was set to 3 (Fig 4), according to the level of membership most genotypes exhibiting two ancestors (61 out of 123) were located in branch A1, whereas genotypes with only one ancestor were distributed in branch A2.1. As for genotypes sharing alleles from three ancestors (32 genotypes), they were evenly distributed along branch B. Moreover with K = 3 the structure analysis divided the population on the basis of fruit size with big fruit (>200gr) belonging to the sub-groups A1.1. and A1.2, while fruit from medium to small size (<100gr) were clustered in the A2 group.

#### Genome-wide association analysis

Associations between SNP alleles and morphological traits were obtained on the basis of a genome-wide association analysis (GWAS) approach using the mixed linear model (MLM) analysis and taking into account the kinship matrix (K) and the population structure matrix Q = 3. As result of GWAS we found a total of 79 significant associations with 15 out of 18 morphological traits evaluated, with the number of associations per trait ranging from one (for FC and PH) to 12 (for FSC and LN) (Table 2). As a whole, these associations corresponded to 36 markers, 12 of which were associated to more than one trait, in general traits related to fruit shape and size, highlighting their common genetic basis. Out of 36 SNPs, 34 belonged to an annotated gene (Solyc) and in three cases more SNPs mapped in the same gene (Solyc01g071770, Solyc10g054010, Solyc11g071530). Most of the SNPs significantly associated to morphological traits mapped on chromosomes 1, 2, 10 and 11 (five, eight, five and 14 markers, respectively), and only one marker mapped to chromosomes 3, 4, 5, and 8, respectively. The percentage of variation explained for each trait (R2) was estimated and ranged from 10% to 33%.

**Table 2:** Markers associated to phenotypic traits by the mixed linear model (MLM). For each marker the position in bp on the related chromosome is reported, together with the corresponding gene (Solyc ID according to SL2.50), the ITAG 2.40 annotation, and the p and R^2^ values.

SolCap ID	Chr	Position	Solyc ID	ITAG 2.40	Traits	p-value	R^2^
**3**	8	60811459	Solyc08g076880	Unknown Protein (AHRD V1)	IT	1.78E-04	0.114
**504**	11	55074586	Solyc11g071670	Pentatricopeptide repeat-containing	ED	1.19E-05	0.130
				protein	FSC	4.72E-05	0.135
					LN	2.39E-04	0.106
					SES	1.69E-04	0.118
**783**	3	3138305	Solyc03g025720	Long-chain-fatty-acid-CoA ligase	FSL	1.24E-04	0.118
**1081**	11	55196715	Solyc11g071840	Calmodulin binding protein	BES	2.06E-04	0.112
					ED	2.51E-08	0.199
					FS	6.82E-06	0.164
					FSC	1.87E-10	0.292
					FW	7.89E-06	0.138
					IT	7.28E-06	0.163
					LN	1.84E-12	0.318
					PI	2.54E-04	0.102
					SES	6.62E-12	0.338
**1253**	2	40037678	Solyc02g070260	Protein phosphatase 1	ED	7.04E-05	0.115
				regulatory subunit 7			
**1981**	10	58672684	Solyc10g074950	Unknown Protein	LN	3.55E-04	0.096
**2032**	2	47612807	Solyc02g084520	Zinc finger transcription factor	LN	4.07E-04	0.096
					SES	1.56E-05	0.151
**2076**	11	54970033	Solyc11g071530	50S ribosomal protein L12-2	ED	1.56E-05	0.126
					FSC	5.30E-06	0.165
					LN	1.19E-05	0.144
					SES	2.41E-05	0.146
**2077**	11	54970111	Solyc11g071530	50S ribosomal protein L12-2	ED	1.56E-05	0.126
					FSC	5.30E-06	0.165
					LN	1.19E-05	0.144
					SES	2.41E-05	0.146
**2119**	10	2347648	Solyc10g008240	Nbs-lrr resi stance protein	PH	1.47E-04	0.110
**2327**	1	1086886	Solyc01g006490	Protein Y di U	BES	3.51E-06	0.169
**2385**	11	3543350	Solyc11g010480	Threonine endopeptidase	PT	3.72E-04	0.104
**2386**	11	3547649	-		PT	3.72E-04	0.104
**2388**	11	3563118	Solyc11g010500	Mitochondrial carrier family	PT	3.72E-04	0.104
**2390**	11	3571600	-		PT	3.72E-04	0.104
**2391**	11	3573991	Solyc11g010520	Unknown Protein	PT	3.72E-04	0.104
**2392**	11	3612093	Solyc11g010560	Kinesin-like protein	PT	3.72E-04	0.104
**2588**	2	53727409	Solyc02g092770	Hydrolase alpha/beta	BES	2.28E-04	0.109
				fold family protein expressed			
**2994**	2	45761358	Solyc02g082030	ABC-type transport system-like	PI	1.35E-04	0.111
**3386**	1	78912845	Solyc01g079760	Mitochondrial carrier protein	FC	3.65E-04	0.101
					FLC	9.21E-05	0.121
**3527**	11	55060751	Solyc11g071640	Beta-D-glucosidase	BES	3.98E-04	0.104
					ED	2.37E-08	0.199
					FS	9.29E-05	0.127
					FSC	1.33E-09	0.270
					FW	1.77E-05	0.129
					IT	1.64E-04	0.119
					LN	1.59E-11	0.302
					SES	8.91E-11	0.310
**3534**	11	55072385	Solyc11g071660	NF-kappa-B-activating protein	ED	1.88E-05	0.121
					FSC	2.55E-05	0.141
					LN	9.27E-05	0.116
					SES	1.01E-04	0.124
**3552**	11	55228352	Solyc11g071900	Self-incompatibility protein	SES	2.12E-04	0.114
				(Fragment)			
**3617**	11	54854070	Solyc11g071340	ABI3-interacting protein2	ED	1.15E-04	0.106
					FSC	1.16E-05	0.154
					LN	1.05E-05	0.146
					SES	6.19E-05	0.133
**3730**	5	4131511	Solyc05g009910	Coiled-coil domain-containing	PI	2.01E-04	0.102
				protein 94			
**4121**	1	94574426	Solyc01g106860	1-phosphatidylinositol 3-kinase	FS	3.78E-04	0.106
**4417**	1	85349943	Solyc01g091770	Ring H2 finger protein	GH	1.58E-04	0.108
**4444**	4	61185790	Solyc04g076250	Unknown Protein	FLC	2.94E-04	0.108
**4597**	2	52417091	Solyc02g090960	Rapid alkalinization factor 3	BES	4.06E-05	0.136
**5624**	2	47148187	Solyc02g083900	Serine/threonine-protein kinase TEL1	BES	3.94E-04	0.104
					ED	8.51E-07	0.164
					FS	3.87E-06	0.174
					FSC	6.65E-06	0.161
					FSL	7.50E-05	0.129
					FW	1.69E-04	0.106
					LN	1.20E-12	0.328
					SES	1.65E-09	0.271
**5625**	2	47218361	Solyc02g083990	Calcium-dependent protein kinase	ED	2.99E-04	0.096
				CPK1 adapter protein 2-like	FSL	5.21E-07	0.198
					LN	5.01E-07	0.188
**5731**	2	45515428	Solyc02g081640	Transcription factort fiiib component	ED	1.57E-04	0.102
					FSC	3.55E-05	0.137
					LN	2.11E-05	0.136
**5987**	10	54449899	Solyc10g054010	BZIP transcription factor	FSC	1.23E-04	0.118
**5993**	10	54449604	Solyc10g054010	BZIP transcription factor	FSC	1.23E-04	0.118
**6954**	1	85349971	Solyc01g091770	Ring H2 finger protein	GH	1.58E-04	0.108
**7469**	10	51524389	Solyc10g051110	NAD dependent	FSC	3.54E-04	0.103
				epimerase/dehydratase			
				family protein			

In order to match the associations with previously identified candidate genes (CGs) for the corresponding traits, a list of CGs was retrieved both from the SOL genomics network(http://solgenomics.net) and from the literature. As a result, 98 genes were selected by merging the two research methods (S3 Table). Eighteen genes were found for fruit colour determination, two for fruit weight, five for plant architecture, two for pericarp thickness, whereas most of them (71 genes) were involved in fruit shape.

**Fig 5:**
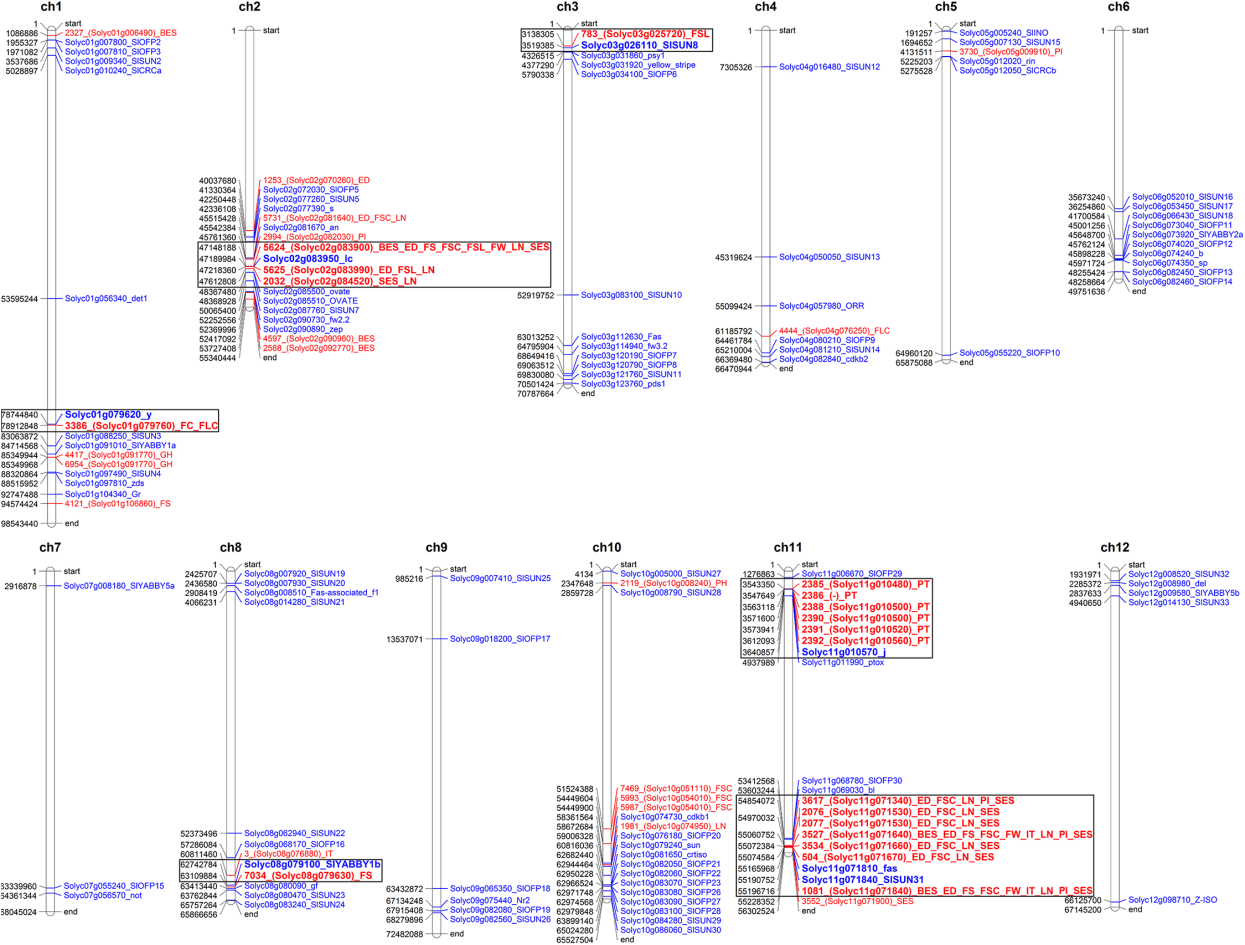
Mapping of markers identified by GWAS and of candidate genes. Physical map of the tomato genome showing the position of the associated SNP markers (in red) and of the candidate genes (in blue). The groups representing a cluster based on LD decay (see S4 Table) are reported in bold type and delimited by a square frame.

Considering the LD-decay distance chromosome by chromosome (S4 Table), it was verified if the SNP-trait associations detected in the present work co-localized with some CGs previously reported, by spotting on the tomato physical map all the CGs and the SNPs associated by GWAS (Fig 5). Overall, six SNP-CG co-localization groups were identified. The most prominent cluster occurred on chromosome 11 where seven SNP markers associated to traits related to fruit shape and size were in LD with the *fasciated* (*fas)* gene (Solyc11g071810). In addition, in the same cluster mapped the SNP marker 1081 that is located into a *sun* gene (Solyc11g071840), validating our methodological approach for mapping. The SNP 1081 represent a transition from C to T in position 55196715 bp on chr 11. The SNP falls in the sixth exon of the gene Solyc11g071840 annotated as Calmodulin binding protein being a member of SUN gene family (SlSUN31). The polymorphism is synonymous, resulting in no changes in the corresponding protein sequence.On the upper arm of the same chromosome, also a cluster harbouring six SNPs associated to PT and the *j* gene (Solyc11g010570) was found. The cluster on chromosome 1 included SNP markers for FC and FLC together with one CG (Solyc01g079620) annotated as *colorless fruit epidermis*, while on chromosome 2 three SNPs (2032, 5624 and 5625)were found associated to fruit shape traits that were in LD with the *lc* gene (Solyc02g083950). On chromosome 3 a co-localization between a marker for FSL (783) and the Solyc03g026110 coding for a SUN protein was found. The last co-localization cluster was identified on chromosome 8 and consisted of marker 7034 associated to FS and Solyc08g079100, a gene annotated as a YABBY family member. Besides these SNP-CG co-localizations, 17 new SNPs associated mostly to fruit morphology were found, pointing out the involvement of new regions of the genome in controlling this trait in tomato in the lower regions of chromosomes 2 and 10.

## Discussion

Phenotypic and genomic data can be used to compare individual genotypes and/or populations with the aim of optimizing characterization, discovery and use of functional allelic variations. In this study, a collection of 123 genotypes was analyzed, which were selected to represent a wide range of phenotypic diversity in tomato. A morphologically based classification mainly regarding fruit traits was carried out, as well as SNP-based genotyping. As expected, the phenotypic clustering did not completely overlap the genetic structure of the population; in fact, it has been previously demonstrated that major phenotypic differences can often occur with only minor genotypic changes [34,35]. A discrepancy between phenotypic characterization and phylogenetic clustering in different tomato collections was already reported in literature [13,36]. In addition, in our study the analysis of morphological traits did not clearly distinguish the predefined groups mainly based on their geographical origin. Based on the morphological traits the accessions were mainly clustered depending on fruit shape. For traits like FC, FSC, SES, LN, and GS, almost all variation was found in the different groups of landraces. At the molecular level, large tomato germplasm collections have been characterized using SSR [37] and SNP markers [18,23,36,38–40] giving insights into population structure, tomato evolutionary history and the genetic architecture of traits of interest [38,41]. In our study, molecular analysis performed by using the SolCAP platform, which includes 7672 SNPs, revealed 87.1% polymorphic markers in the entire collection, but this value decreased to about 50% in the different sub-populations, thus showing different pattern of segregation for each sub-group. The percentage of polymorphic SNPs in our tomato collection is similar to that reported in previous studies carried out using the same genotyping platform in different tomato collections [23, 36].

To evaluate the level of genetic diversity in the entire collection and within different groups of germplasm the observed and expected heterozygosity, and polymorphism information content were measured. We found that the level of heterozygosity was low, as expected in tomato, but variable among the sub-populations. The analysis of nucleotide diversity pattern showed that CVs maintained the largest amount of diversity within the collection, as also revealed by the STRUCTURE model-based clustering (for K = 3), in which most of the ILs showed one or two ancestors, while CVs derived from three ancestors. Indeed, genetic diversity was lower in the landraces compared to the contemporary cultivars, probably due to the different breeding programs that these two categories underwent. The long history of crossing cultivars to wild relatives has broadened the genetic diversity in contemporary germplasm with respect to vintage and landrace germplasm [23,41], despite of a lower phenotypic diversity due to a long breeding work aimed at increasing uniformity of shape and weight [38]. The lower genetic diversity estimated for all the three sub-populations of tomato landraces (American, Italian and Other) was in line with data reported by [23,42] in case of Latin American landraces and by [9] in traditional landraces from the Old-World. This was probably due to the fact that farmers are often used to collect seeds from best fruits and, rather than selecting the more productive genotypes, they prefer to maintain a good fruit quality [9]. By contrast, data from [13] revealed a high level of molecular diversity in landraces compared to tomato modern cultivars. These contrasting results are probably due to differences in the germplasm collection and molecular markers sampled for the analysis.

In the present study, we used a high-throughput genotyping platform to characterize our collection of genotypes and to verify that the genetic variability available in this collection was suitable to perform an informative association mapping approach. To this purpose, we used as case of study traits related to plant architecture and fruit morphology, which are reportedly stable and not highly influenced by environmental conditions [13,18]. A few GWASs have been undertaken in the last few years in tomato [24,40,43,44] For this species, the linkage disequilibrium decays over large genomic regions making the identification of causal polymorphism responsible for phenotypic variations the main limit of this approach. Despite this, good results were obtained thanks to the availability of improved statistical methods (as the MLM model, [45]) and more cost-effective technologies for genotyping. The GWAS scan we carried out revealed a total of 36 markers associated with the variation of 15 traits, allowing the identification of previously known as well as novel loci. Of the 36 detected markers, 30 were associated to fruit morphology traits and were mostly localized on chromosomes 2, 10 and 11. On these three chromosomes QTLs for the traits analysed in our study were also previously mapped [46]. Due to LD, the SNPs identified often co-localized and this has been observed in other GWAS studies in tomato [43,44] and other species [47,48]. In some cases, the same SNP was associated to different traits, as was the case of marker 1081 that was associated to nine traits on chromosome 11. Markers associated to several traits may easily be explained with the high correlation existing among phenotypic descriptors of the fruit ([13], this study) or to pleiotropic effects.

In addition to the 36 SNP markers found associated to traits in our GWAS analysis, we mapped on the 12 tomato chromosomes 98 CGs previously identified for the traits analyzed. Altogether, we detected 16 chromosomal regions where at least 4 genes and/or markers clustered. In some cases, these regions consisted of only CGs or associated SNPs. We first focused our attention on six regions where the co-localization of CGs and SNPs was evidenced, since these validated the trait-SNP association detected in our study. Among these, five co-localizations related to fruit morphology determinants (i.e. ED, FS, FSL, BES, SES, LN, PT) mapped to the upper regions of chromosomes 3 and 11, and to the lower regions of chromosomes 2, 8, 11, where also QTLs for these traits were previously located. The remaining co-localization of CGs and associated SNPs was related to the fruit colour (FC and FLC), and is located to the lower part of chromosome 1.

Despite the tremendous diversity of fruit shape in tomato, these are explained to a large extent by mutations in four genes, which are *sun*, *ovate*, *lc* and *fas* [49]. Among these, mutations of *sun* and *ovate* confer elongated fruit shape, whereas *lc* and *fas* control locule number, and if mutated, confer fruit fasciation and flat shape. These genes map to chromosomes 2 (*lc* and *ovate*), 10 (*sun*) and *11* (*fas*). The tomato fruit shape genes *sun*, *o* and *fas* belong to IOD/SUN, Ovate Family Protein (OFP) and YABBY gene family, respectively. Huang and co-workers [50] identified34SlSUN, 31 SlOFP and 9 SlYABBY genes in tomato and mapped their position on the 12 chromosomes. So far, we report the position of all these genes in our map besides the SNPs associated markers. In addition, very recently another gene (*elf1*) influencing elongation of tomato fruit was mapped on the lower arm of chromosome 8 between *SlSUN23* and *SlSUN24* [51]. The association cluster on chromosome 2 includes the *lc* gene (Solyc02g083950) and three SNP markers (2032, 5624 and 5625) associated to various traits. *LC* is a WUSCHEL homeodomain protein that controls the number of carpel primordia and its mutation results in a fruit with more than the typical two or three locules [52,53]. Increases in locule number often lead to a flat fruit of a larger size; this mutation is therefore common in beefsteak tomato [49,53]. On chromosome 8, the strongest association for FS (*p* = 8.02E-5) was evidenced for SNP 7034 in LD with the *SlYABBY1b* gene (Solyc08g079100).YABBY family proteins are involved in the control of locule number and also in the number of all floral organs [54]. *SlYABBY1b* is expressed in young floral buds [50] confirming a role in early reproduction and gynaecium patterning. van der Knapp and collaborators [55] showed that due to the function of YABBY family proteins and their expression pattern, *fas* was hypothesized to control the final fruit size. The position of our associated marker laid down between the two markers flanking the *elf1* gene reported by [51] thus reinforcing the role of this novel candidate gene in affecting fruit shape. Finally, on chromosome 11 we evidenced the most numerous cluster composed by seven SNP markers (3617, 2076, 2077, 3527,3534, 504, and 1081) associated to nine traits and two genes, FAS (Solyc11g071810) and *SUN31* (Solyc11g071840). The former gene was already aforementioned for its involvement in fruit shape and size. *SUN31*, as other SUN family members, is expressed during flower and fruit development supporting a possible role in the definition of the final fruit shape. Previously described mutations of SUN change fruit shape by redistributing fruit mass; an increase in cells in the proximal-distal direction is accompanied by a decrease in cell number in the columella and septum in the medio-lateral direction throughout the entire fruit [56].

Finally, six SNP markers associated to the PT trait (2385, 2385, 2386, 2388, 2390, 2391, 2392) were found on chromosome 11, in LD with the *JOINTLESS* (*J*) gene (Solyc11g010570) that encodes for a MADS box transcription factor. It might play a role in floral meristem identity rather than fruit development leading to heavier fruit with less seed [57]. Whether the association between markers on the long arm of chromosome 11, the trait PT and the locus *J* indicates a role for *J* in pericarp development or the existence of a different linked causal gene will require further investigation. The possibility of a spurious association also exists because the *j* trait is generally introgressed in modern processing cultivars that, on the other hand, have also been bred for improved pericarp thickness.

Since it is widely ascertained that the effect of major genes on fruit size and shape also depends on the genetic background in which they are active, the identification of modifier genes on these traits is still a challenge, as demonstrated by the discovery of two genes (*sov1* and *sov2*) suppressing the *ovate* effect of fruit shape [18]. In our case, besides the 19 SNP markers in LD with CGs, other 17 SNPs involved in the genotype/phenotype associations discovered mapped to genomic regions where no CGs related to the traits had been reported. One marker for fruit shape (marker 4121) on the long arm of chromosome 1 does not co-localize with CGs but resides in a chromosomal region where other genes putatively related to fruit shape were mapped, corresponding to the previously reported QTL fs1.b [58]. This could reinforce the hypothesis that some minor genes might affect fruit shape, beside the action of the well-known major genes. Also, the identification of the additional fruit weight QTL fw11.3 revealed the existence of new regulators in fruit weight [59], besides the two major genes *fw2*.*2* and *fw3*.*2*. Our analysis also confirmed the involvement of this chromosomal region in fruit weight determination, where two markers associated to FW mapped. Similarly, beside the action of the major gene *SELF-PRUNING* (*SP*), a minor effect on growth habit might be due to genes located on the lower arm of chromosome 1, as well as other putative regions affecting growth habit were previously mapped by [13] on chromosomes 5, 8 and 11.

Comprehensively, all novel markers here associated to the 15 traits have been identified only thanks to the extent of the genetic variability available in our heterogeneous germplasm collection and made accessible by GWAS approaches. These SNPs, if appropriately validated, could be adopted as potent selection markers for marker-assisted selection in tomato breeding.

## Conclusions

The phenotypic characterization of our tomato collection showed higher morphological variation in landraces compared to cultivars, while opposite results were obtained from the genotypic analysis, whereby the cultivars maintained the largest amount of diversity in the collection.

A total of 7,720 SNP loci were genotyped in 123 tomato lines, and the GWAS approach revealed 36 markers associated to the variation of 15 traits. Of the 36 significant SNPs, 30 were associated to fruit morphology, including traits such as pericarp thickness and fruit weight. Our results confirmed the strong involvement of genomic regions of chromosomes 2 and 11 in determining fruit shape and contributed to a better understanding of minor genes underlying fruit shape determination in the cultivated germplasm of tomato.

Finally, thanks to the wide diversity captured by our collection we were able to detect new marker/trait associations, overall for pericarp thickness and fruit morphology, which can provide viable indirect selection tools in a practical breeding program. The same approach would be in the future exploited for targeting additional traits, thanks to a further effort of phenotyping our collection for desirable traits to improve tomato.

## Supporting Information

S1 Fig PCA analysis of the tomato collection. Principal component analysis carried out based on the morphological traits.
(PPT)

S1 File NCBI Accession Numbers. (XLS)

S1 Table List of genotypes used with measures of their morphological characteristics. For each genotype, the source providing seeds, the accession number from the source, the common name (if available) and the geographical origin are reported. Acronym and description of morphological trait measures are reported in the legend below.
(XLSX)

S2 Table Correlation matrix of the 18 morphological traits. (PPTX)

S3 Table List of candidate genes involved in the determination of the morphological traits under study. (XLSX)

S4 Table Linkage Disequilibrium decay on each chromosome. (XLS)

## Abbreviations

AL: American landraces
BES: Blossom-end shape
CG: Candidate gene
CV: Cultivars
ED: Equatorial diameter
FC: Fruit colour
FLC: Fruit flesh colour
FS: Fruit-shape index
FSC: Fruit-shape cross section
FSL: Fruit-shape longitudinal section
FW: Fruit weight
GH: Growth habit
GS: Green shoulder
IL: Italian landraces
IT: Inflorescence type
LN: Locule number
OL: Other landraces
PD: Polar diameter
PH: Plant height
PI: Pericarp tickness index
PT: Pericarp thickness
PUF: Puffiness
SES: Stem-end shape
WS: Wild species
