# Supplementary material for: Exploring a Tomato Landraces Collection for Fruit-Related Traits by the Aid of a High-Throughput Genomic Platform
Source: PLoS One. 2015 Sep 22;10(9):e0137139. doi: 10.1371/journal.pone.0137139 (PMC4579088; doi:10.1371/journal.pone.0137139)
Supplement: S1 Fig — Principal component analysis carried out based on the morphological traits. (PPT) [file pone.0137139.s001.ppt]

## Slide 1
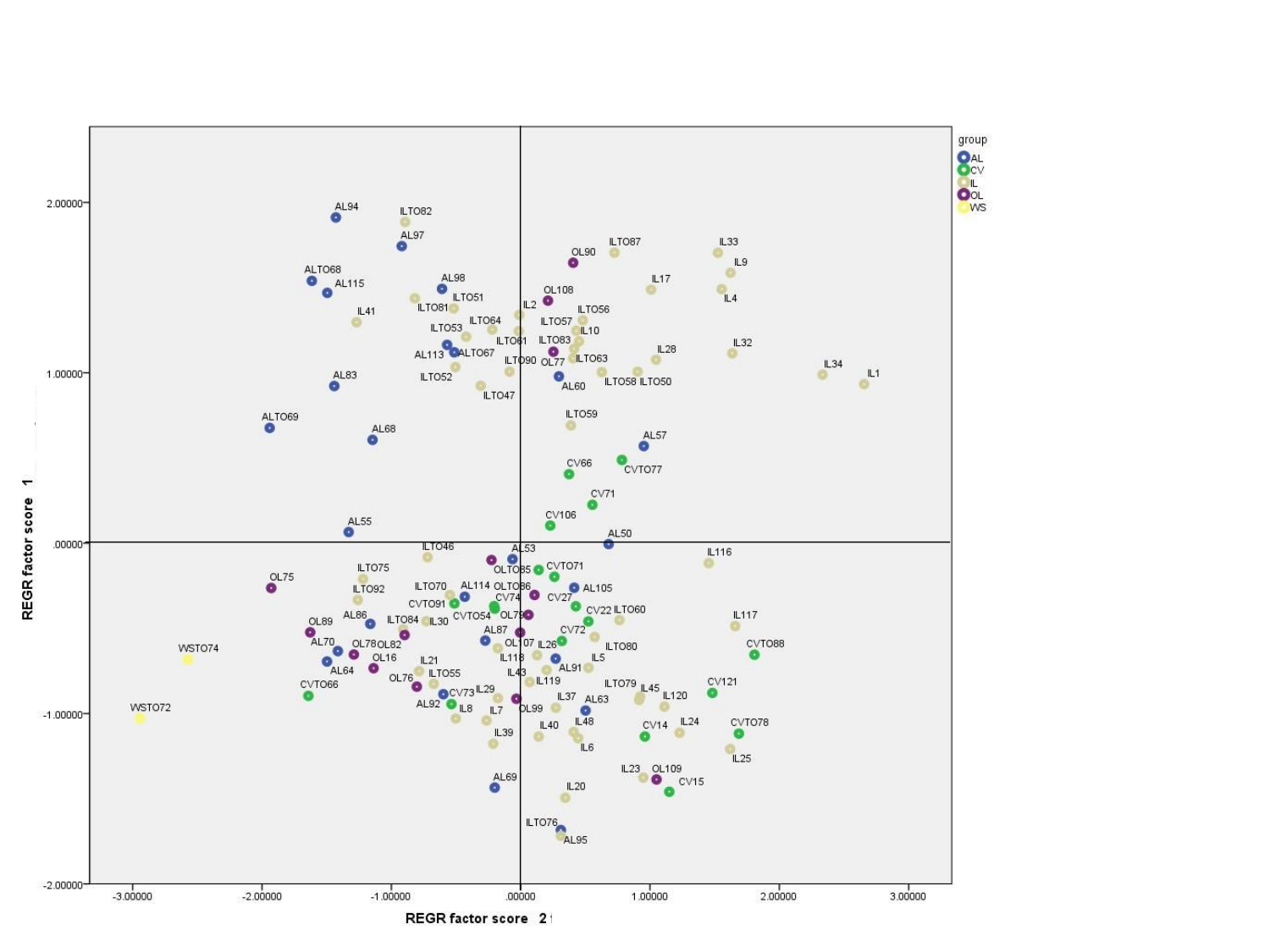

## Slide 2
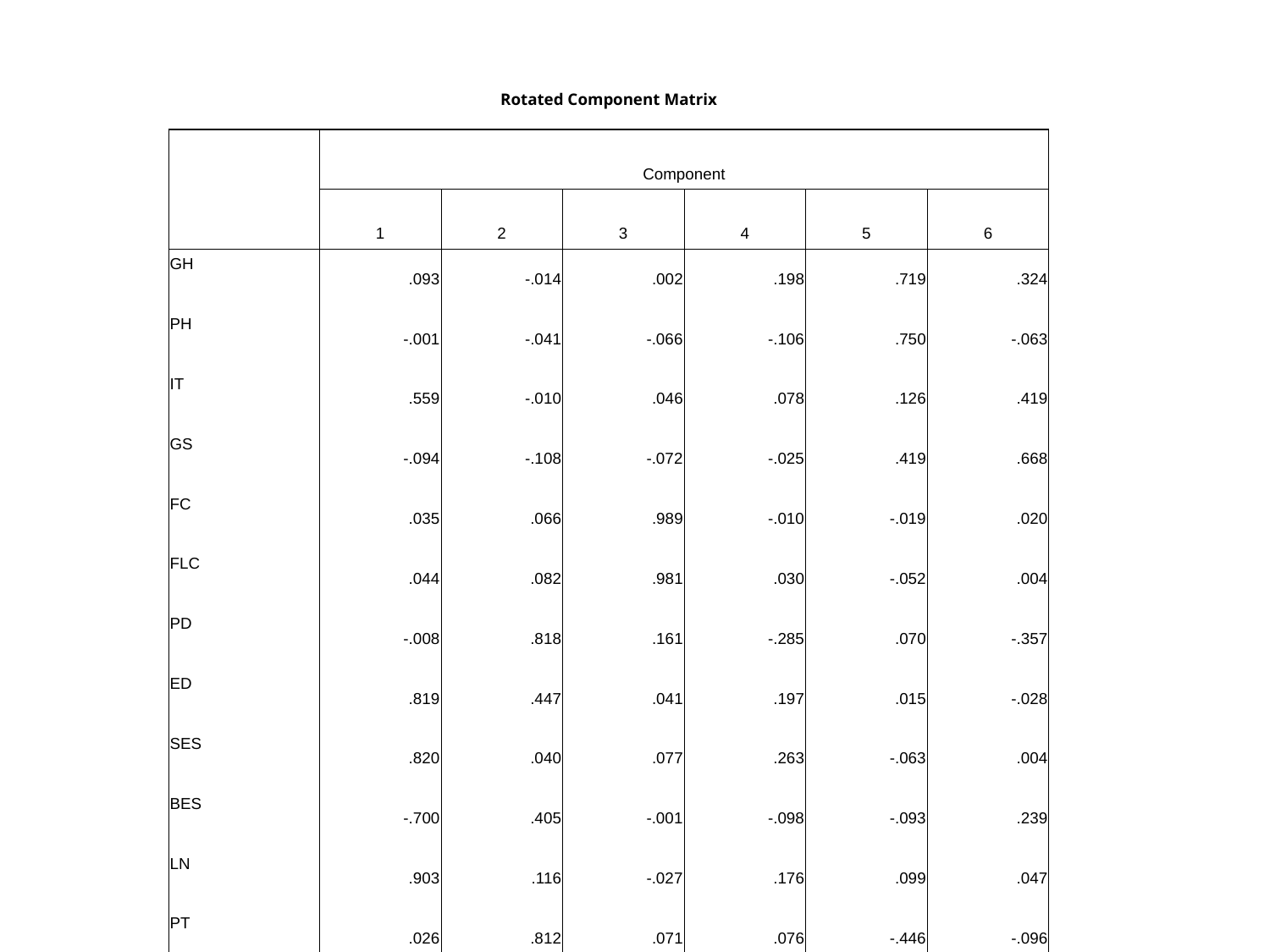

| Rotated Component Matrix | | | | | | | |
| --- | --- | --- | --- | --- | --- | --- | --- |
| | Component | | | | | | |
| | 1 | 2 | 3 | 4 | 5 | 6 | |
| GH | .093 | -.014 | .002 | .198 | .719 | .324 | |
| PH | -.001 | -.041 | -.066 | -.106 | .750 | -.063 | |
| IT | .559 | -.010 | .046 | .078 | .126 | .419 | |
| GS | -.094 | -.108 | -.072 | -.025 | .419 | .668 | |
| FC | .035 | .066 | .989 | -.010 | -.019 | .020 | |
| FLC | .044 | .082 | .981 | .030 | -.052 | .004 | |
| PD | -.008 | .818 | .161 | -.285 | .070 | -.357 | |
| ED | .819 | .447 | .041 | .197 | .015 | -.028 | |
| SES | .820 | .040 | .077 | .263 | -.063 | .004 | |
| BES | -.700 | .405 | -.001 | -.098 | -.093 | .239 | |
| LN | .903 | .116 | -.027 | .176 | .099 | .047 | |
| PT | .026 | .812 | .071 | .076 | -.446 | -.096 | |
| PUF | .112 | .285 | -.105 | .148 | .187 | -.545 | |
| FS | -.644 | .366 | .132 | -.392 | .046 | -.317 | |
| FW | .679 | .637 | .024 | .043 | .094 | -.035 | |
| PI | -.567 | .193 | -.084 | .113 | -.554 | .251 | |
| FSL | -.228 | .005 | -.012 | -.894 | .025 | .064 | |
| FSC | .722 | .038 | .040 | .163 | .002 | -.233 | |
| TP | .390 | -.090 | .023 | .844 | .015 | -.048 | |
| | | | | | | | |
| | | | | | | | |
