# Supplementary material for: Exploring a Tomato Landraces Collection for Fruit-Related Traits by the Aid of a High-Throughput Genomic Platform
Source: PLoS One. 2015 Sep 22;10(9):e0137139. doi: 10.1371/journal.pone.0137139 (PMC4579088; doi:10.1371/journal.pone.0137139)
Supplement: S2 Table — (PPTX) [file pone.0137139.s004.pptx]

## Slide 1
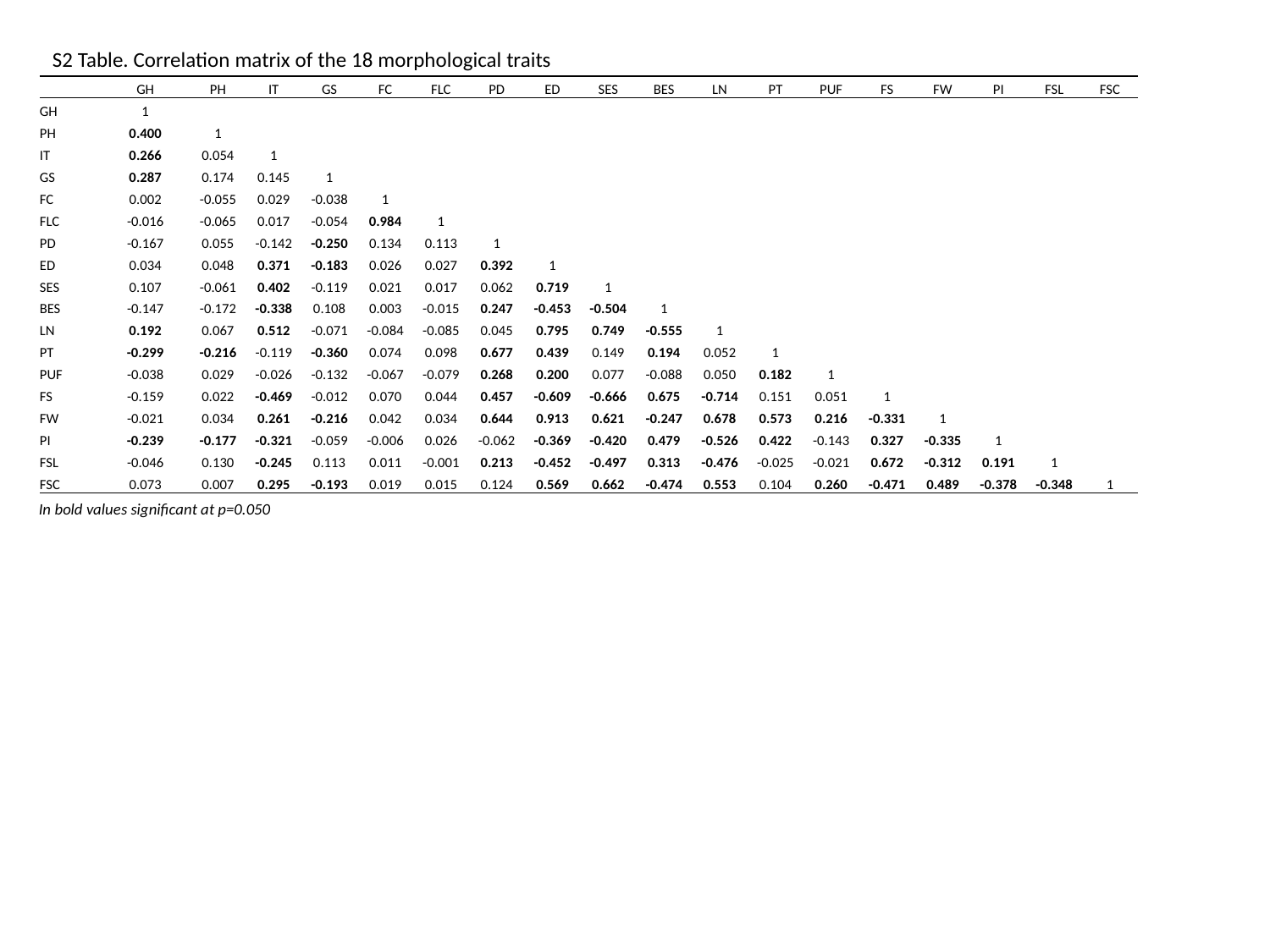

S2 Table. Correlation matrix of the 18 morphological traits
| | GH | PH | IT | GS | FC | FLC | PD | ED | SES | BES | LN | PT | PUF | FS | FW | PI | FSL | FSC |
| --- | --- | --- | --- | --- | --- | --- | --- | --- | --- | --- | --- | --- | --- | --- | --- | --- | --- | --- |
| GH | 1 | | | | | | | | | | | | | | | | | |
| PH | 0.400 | 1 | | | | | | | | | | | | | | | | |
| IT | 0.266 | 0.054 | 1 | | | | | | | | | | | | | | | |
| GS | 0.287 | 0.174 | 0.145 | 1 | | | | | | | | | | | | | | |
| FC | 0.002 | -0.055 | 0.029 | -0.038 | 1 | | | | | | | | | | | | | |
| FLC | -0.016 | -0.065 | 0.017 | -0.054 | 0.984 | 1 | | | | | | | | | | | | |
| PD | -0.167 | 0.055 | -0.142 | -0.250 | 0.134 | 0.113 | 1 | | | | | | | | | | | |
| ED | 0.034 | 0.048 | 0.371 | -0.183 | 0.026 | 0.027 | 0.392 | 1 | | | | | | | | | | |
| SES | 0.107 | -0.061 | 0.402 | -0.119 | 0.021 | 0.017 | 0.062 | 0.719 | 1 | | | | | | | | | |
| BES | -0.147 | -0.172 | -0.338 | 0.108 | 0.003 | -0.015 | 0.247 | -0.453 | -0.504 | 1 | | | | | | | | |
| LN | 0.192 | 0.067 | 0.512 | -0.071 | -0.084 | -0.085 | 0.045 | 0.795 | 0.749 | -0.555 | 1 | | | | | | | |
| PT | -0.299 | -0.216 | -0.119 | -0.360 | 0.074 | 0.098 | 0.677 | 0.439 | 0.149 | 0.194 | 0.052 | 1 | | | | | | |
| PUF | -0.038 | 0.029 | -0.026 | -0.132 | -0.067 | -0.079 | 0.268 | 0.200 | 0.077 | -0.088 | 0.050 | 0.182 | 1 | | | | | |
| FS | -0.159 | 0.022 | -0.469 | -0.012 | 0.070 | 0.044 | 0.457 | -0.609 | -0.666 | 0.675 | -0.714 | 0.151 | 0.051 | 1 | | | | |
| FW | -0.021 | 0.034 | 0.261 | -0.216 | 0.042 | 0.034 | 0.644 | 0.913 | 0.621 | -0.247 | 0.678 | 0.573 | 0.216 | -0.331 | 1 | | | |
| PI | -0.239 | -0.177 | -0.321 | -0.059 | -0.006 | 0.026 | -0.062 | -0.369 | -0.420 | 0.479 | -0.526 | 0.422 | -0.143 | 0.327 | -0.335 | 1 | | |
| FSL | -0.046 | 0.130 | -0.245 | 0.113 | 0.011 | -0.001 | 0.213 | -0.452 | -0.497 | 0.313 | -0.476 | -0.025 | -0.021 | 0.672 | -0.312 | 0.191 | 1 | |
| FSC | 0.073 | 0.007 | 0.295 | -0.193 | 0.019 | 0.015 | 0.124 | 0.569 | 0.662 | -0.474 | 0.553 | 0.104 | 0.260 | -0.471 | 0.489 | -0.378 | -0.348 | 1 |
In bold values significant at p=0.050
